# ﻿A new species of *Cotoneaster* (Rosaceae) from western Sichuan, China

**DOI:** 10.3897/phytokeys.236.111819

**Published:** 2023-11-27

**Authors:** Mingwan Li, Dan Li, Mengfei Lu, Shuangfeng Mo, Shen Ding, Yuanyuan Chen, Yong Lai, Dangquan Zhang, Wenbo Liao, Qiang Fan

**Affiliations:** 1 College of Forestry, Henan Agricultural University, Zhengzhou 450002, China Henan Agricultural University Zhengzhou China; 2 State Key Laboratory of Biocontrol and Guangdong Provincial Key Laboratory of Plant Resources, School of Life Sciences, Sun Yat-sen University, Guangzhou 510275, China Sun Yat-sen University Guangzhou China

**Keywords:** Anatomical, chloroplast genome, leaf epidermis, palynological, Ser. *Salicifolii*

## Abstract

*Cotoneasterdensiflorus*, a new species of Rosaceae from western Sichuan, China, is described and illustrated. Morphologically, we inferred that the new species belongs to CotoneasterSer.Salicifolii sensu Yü et al. (1974) in the Flora of China and Fryer and Hylmö (2009). This species is most similar to *C.salicifolius*, but differs in its leaf blade of ovate-lanceolate to obovate shape (vs. elliptic-oblong to ovate-lanceolate), smaller length-width ratio of 2.37 ± 0.31 (vs. 3.17 ± 0.32), slightly conduplicate (vs. not conduplicate), less lateral veins of 6–8 pairs (vs. 12–16 pairs), upper surface slightly rugose (vs. rugose), leaf margin plane (vs. revolute), lower surface densely grey tomentose (vs. grey tomentose, with bloom), greater corolla diameter of 7–9 mm (vs. 5–6 mm), styles 2 (vs. 2–3), pyrenes 2 (vs. 2–3), larger pollen grains P/E values of 2.05 ± 0.12 (vs. 1.19 ± 0.05) and leaf epidermis type W (vs. type I). Based on phylogenetic analysis of the whole chloroplast genome, *C.densiflorus* is sister to *C.rhytidophyllus*, but distantly related to *C.salicifolius*.

## ﻿Introduction

*Cotoneaster* Medik. (Rosaceae, Maloideae) is a morphologically highly variable genus that is naturally distributed in Europe, North Africa and the temperate areas of Asia except Japan. The Himalayas and neighboring mountains in Yunnan and Sichuan of China are species diversity and distribution centers for this genus (Fryer and Hylmö 2009). Due to the frequent occurrence of hybridization and polyploidisation, together with apomixis, its infrageneric classification is controversial and unstable (Campbell et al. 2007; Lo and Donoghue 2012; Li et al. 2014, 2017a; Meng et al. 2021). The number of species in the genus ranges from about 50 to more than 400 according to different (and often contradictory) species concepts and traits (Flinck and Hylmö 1966; Yü et al. 1974; Phipps et al. 1990; Lu et al. 2003; Fryer and Hylmö 2009; Dickore and Kasperek 2010). In the Flora of China, the genus was divided into three sections (Sect. Densiflos, Sect. Cotoneaster and Sect. Uniflos), based on the number of flowers in the inflorescence (Yü et al. 1974). However, more studies supported the conclusion of two major sections/subgenera: *Chaenopetalum* and *Cotoneaster* (Koehne 1893; Flinck and Hylmö 1966; Phipps et al. 1990; Fryer and Hylmö 2009), which was also proved by molecular phylogenetic studies, based on nuclear ITS, chloroplast or low-copy nuclear genes in the last decade (Li et al. 2012, 2014; Lo and Donoghue 2012; Meng et al. 2021). For species in Subg. Chaenopetalum, the flowers in a cyme open simultaneously, within white spreading (rarely pink) petals, white filaments and purple to black anthers. While for species in Subg. Cotoneaster species, the flowers in a cyme open continuously over an extended period, within red (rarely pink) erect (less suberect) petals, red, pink or white filaments and white anthers.

Within the sections/subgenera, the further division into 7–39 series was mainly based on morphology of stems, branches, leaves, number of pyrenes and resistance (Flinck and Hylmö 1966; Yü et al. 1974; Phipps et al. 1990; Fryer and Hylmö 2009). The Ser. Salicifolii included 5–7 species and about three varieties, whose name was derived from the mostly lanceolate and willow-like leaves. In Fryer and Hylmö (2009) and Flora of China (Yü et al. 1974), the important morphological features that distinguished the Ser. Salicifolii species from other series include evergreen or semi-evergreen shrub, leaf blade leathery, mostly lanceolate, abaxially persistently densely tomentose, veins impressed, white spreading petals, and pyrenes 2–4 (–5). Multiple phylogenetic trees of *Cotoneaster*, based on low-copy nuclear and chloroplast genes showed that these series, as smaller taxonomic units under subgenera, were not monophyletic (Lo and Donoghue 2012; Meng et al. 2021), which agreed with our recent phylogenetic study results on Ser. Salicifolii (unpublished study).

During our field survey in western Sichuan Province, an interesting population that shares morphological affinities with Ser. Salicifolii species was discovered. These affinities are based on evergreen or semi-evergreen shrub, leathery leaf blade, dense inflorescence and white spreading petals. However, this taxon was not completely similar to any species that has been described worldwide. Furthermore, the individuals of this species were distributed in Baoxing County, which is located in Siguniang and Jiajin Mountains at the eastern edge of the Hengduan Mountains, a biodiversity hotspot in southwest China (Zhang and Ma 2008, 2009; Li et al. 2017b). Interestingly, this county was also the type specimen collection site of *C.salicifolius* and *C.moupinensis*. Over the past 10 years, approximately 10 new plant species were discovered and illustrated in Baoxing County, such as *Youngiabaoxingensis* Y.S. Chen (Chen 2018), *Primulaluteoflora* X.F. Gao & W.B. Ju (Ju et al. 2018), *Berberisjinwu* Y.K. Li, Harber, Y.W. Xing & C.C. Yu (Li et al. 2022) and several others. From 2016 to 2023, after detailed morphological examination, specimen collection and comparison with herbarium specimens sampled in this region, we identified this shrub as a new species and determined its phylogenetic position in the genus of *Cotoneaster* through whole chloroplast genome data. Our study not only enriched the diversity of *Cotoneaster* species in China, but also highlighted the importance of the basic survey of biodiversity in this area of Sichuan and the Hengduan Mountains.

## ﻿Material and methods

Plant morphological features and habits were recorded and photographed in the field during the flowering and fruiting periods of the putative new species. The characteristics and measurements were compared with those of its related species naturally occurring in Sichuan Province (i.e. *C.salicifolius* and *C.rhytidophyllus*) as described in the Flora of China (Yü et al. 1974) and related taxonomic literature of *Cotoneaster* (Fryer and Hylmö 2009). Voucher specimens were deposited in the Herbarium of SunYat-sen University (SYS) in China.

For scanning electron microscopy (SEM) observations, pollen grains of this putative new species and two related species were collected from specimens (*M.W. Li 20230617007*, *Q.Fan 15682-01* and *Q.Fan 15643* (SYS)). For scanning electron microscopy (SEM) observation, the pollen grains were transferred onto metal stubs with double-sided adhesive tape and sputter-coated with technical gold (Li et al. 2017b; Xiong et al. 2019). Approximately 20 randomly selected pollen grains were scanned and photographed by the SEM (S-3400, Hitachi, Tokyo, Japan) at 5 kV accelerating voltage and SE detector, then the pollen grains were measured and the polar axis (P), equatorial axis (E) and the ratio of polar axis length to equatorial axis length (P/E) were calculated. The nomenclature for pollen morphology mainly followed Erdtman (1943, 1952) and the terminology of ornamentation mainly followed Ueda and Tomita (1989).

Leaf epidermal materials were prepared from mature leaves and macerated in 1:1 (by volume) hydrogen dioxide solution and glacial acetic acid and then were boiled in a water bath for 1.5–2 h. After being rinsed with water, leaf materials were transferred to Schultze’s solution for 30 minutes. Finally, pieces of leaf epidermis were stained with a solution of 1% safranin prior to mounting in glycerine gel. Prepared cuticles were observed using a SY100 light microscope and JSM-6330F SEM. The nomenclature of stomatal types and leaf epidermis is mainly based on the descriptions of Wilkinson (1979), Prabhakar (2004) and Ding et al. (2008).

Total genomic DNA was extracted using the Plant Genomic DNA Kit (DP305, Tiangen Biotech Co., Ltd., Beijing, China) and DNA quality was measured using a NanoDrop 2000 spectrophotometer (NanoDrop Technologies; Thermo Fisher Scientific, Inc., Wilmington, DE, USA). The qualified DNAs (≥50 ng) were sent to Novogene Bioinformatics Technology Co., Ltd. (Beijing, China) for paired-end (PE) library construction and genome-skimming sequencing. The generated reads were assembled by the GetOrganelle (Jin et al. 2020) pipeline. In this pipeline, the chloroplast genome of *C.salicifolius* (KY419943.1; Zhang et al. (2017)) was set as a reference. The genome annotation was performed with CpGAVAS (Liu et al. 2012), then the inverted repeat (IR) boundaries were manually adjusted and confirmed on geneious prime2023.0.4 (https://www.geneious.com/). In order to determine the phylogenetic position of this species in *Cotoneaster*, complete chloroplast genomes of 64 accessions downloaded from NCBI and seven unpublished Ser. Salicifolii taxa were obtained to reconstruct the phylogenetic trees with *Rhaphiolepisbibas* and *Rhaphiolepisprinoides* as outgroups (Table 1). The sequences were aligned using MAFFT version 7 (https://mafft.cbrc.jp). The best-fit nucleotide substitution model was determined by ModelFinder (Kalyaanamoorthy et al. 2017). The maximum-likelihood (ML) phylogenetic tree was constructed using RAxML-HPC Blackbox Software (Stamatakis 2014) with the GTRGAMMAI model and 1000 bootstrap replicates to assess the support for each branch. Bayesian inference (BI) was conducted using MrBayes v.3.2.7 (Ronquist et al. 2012) with Markov chains for at least 10,000 generations and sampled every 10 generations. After the average standard deviation of split frequencies (ASDFs) was assessed and reached < 0.01, the first 25% trees were discarded as burn-in.

**Table 1.** Taxa, voucher information, and GenBank accession numbers of the chloroplast genome sequences used in this study (a. This study, b. Meng et al. 2021, c. Liu et al. 2020, d. Chen et al. 2022, e. unpublished study).

Taxon	Voucher	Accession numbers
**CotoneasterSubg.Chaenopetalum**		
*Cotoneasterdensiflorus*	14924	OR478167 ^a^
*C.argenteus*	13466-1	MK578683 ^b^
*C.astrophoros*	17073	MK650065 ^b^
*C.conspicuus*	15902	MK638987 ^b^
	17912	MK650062 ^b^
*C.coriaceu*s	13462-12	MK650049 ^b^
	13462-10	MK561974 ^b^
	–	NC_060440 ^b^
*C.dammeri* spp. *songmingensis*	17091	MK605511 ^b^
*C.delavayanus*	17148-5	MK605518 ^b^
*C.fulvidus*	17168	MK614792 ^b^
*C.glaucophyllus*	15960-1	MK561976 ^b^
*C.hebephyllus*	14669	MK638988 ^b^
*C.lacteus*	17153-5	MK605517 ^b^
*C.marginatus*	17082	MK605510 ^b^
*C.multiflorus*	YZSP	MK650060 ^b^
*C.pannosus*	16009	MK605509 ^b^
*C.rockii*	17155-5	MK605515 ^b^
*C.salicifolius*	16911-2	MK638989 ^b^
	–	NC_060455 ^b^
C.salicifoliusvar.henryanus	2241	MN577863 ^c^
*C.serotinus*	15962-2	MK578685 ^b^
*C.sherriffii*	17178-2	MK614794 ^b^
*C.soongoricus*	ZGE-1	MK650057 ^b^
*C.submultiflorus*	MYS-1	MK650061 ^b^
*C.turbinatus*	16900	MK650054 ^b^
*C.vandelaarii*	17186-1	MK544858 ^b^
*C.angustus*	14996	–^e^
*C.coriaceus*	B15184	–^e^
*C.hylmoei*	15219	–^e^
*C.rhytidophyllus*	15661	–^e^
*C.rugosus*	15270	–^e^
*C.turbinatus*	B15045	–^e^
*C.glabratus*	14989	–^e^
**CotoneasterSubg.Cotoneaster**		
*Cotoneasteracuminatus*	324-64*B	MK650045 ^b^
*C.acutifolius*	13755-27	MK638990 ^b^
*C.adpressus*	12388	MK638993 ^b^
*C.affinis*	14662-06	MK650051 ^b^
*C.bullatus*	17157-8	MK614791 ^b^
*C.* cf_*chengkangensis*	17145	MK638992 ^b^
	17145-1	MK605514 ^b^
*C.cinerascens*	17136-1	MK638991 ^b^
*C.cochleatus*	14835	MK524400 ^b^
*C.dielsianus*	15959-2	MK614800 ^b^
*C.foveolatus*	860-84*E	MK650046 ^b^
*C.franchetii*	17191	MK638985 ^b^
*C.frigidus*	14650-10	MK561975 ^b^
*C.gamblei*	14663-09	MK650052 ^b^
*C.horizontalis*	1981-65	MK561973 ^b^
*C.huahongdongensis*	17187-8	MK614796 ^b^
*C.integerrimus*	1234*82C	MK614799 ^b^
*C.langei*	17181-2	MK605516 ^b^
*C.leveillei*	17122-8	MK544857 ^b^
*C.melanocarpus*	13756-19	MK561977 ^b^
*C.microphyllus*	17028_25	MK544856 ^b^
*C.moupinensis*	628*97C	MK614797 ^b^
*C.obscurus*	1231-82*C	MK614798 ^b^
*C.perpusillus*	PZXY4-8	MK638994 ^b^
*C.praecox*	Cnanshan	MK638986 ^b^
*C.qungbixiensis*	17138-1	MK605513 ^b^
*C.reticulatus*	WMXZ	MK650055 ^b^
*C.rotundifolius*	17029-1	MK650063 ^b^
*C.rubens*	17175-1	MK614793 ^b^
*C.schantungensis*	SD1	MK650053 ^b^
*C.shansiensis*	SS-1	MK650064 ^b^
*C.subadpressus*	17167	MK650058 ^b^
*C.tenuipes*	7276*C	MK650047 ^b^
*C.vellaeus*	17179-7	MK614795 ^b^
*C.verruculosus*	17137-1	MK605512 ^b^
*C.villosulus*	13165*B	MK650048 ^b^
*C.zabelii*	XB3	MK650056 ^b^
**Outgroups**		
*Rhaphiolepisbibas*	201819	MN577877 ^c^
*R.prinoides*	–	MT876398 ^d^

Flow cytometry was used to estimate the genome size and to determine the ploidy level. Samples were prepared by a modified method according to Rothleutner et al. (2016). Approximately 1 cm^2^ of fresh leaf tissue was chopped with maize as the internal standard using a blade in cold (4 °C) Otto I buffer (0.1 M citric acid, 0.5% (v/v) Tween 20) for 90 s before being filtered through a 50-μm nylon mesh. The suspension was stained with Otto II buffer (0.4 M Na_2_HPO_4_·12H_2_O), β-mercaptoethanol, Rnase and PI fluorochrome. An Accuri C6 flow cytometer (BD Biosciences, San Jose, CA, USA) equipped with 488-nm laser, was employed with a sample flow rate 14 µl min^−1^. Fluorescence measurements were obtained using the FL2 (585/40 nm) optical filter, capturing 10 000 events and utilizing the FL2-A values for the 2C peak.

## ﻿Result and discussion

### ﻿Taxonomic treatment

#### ﻿ Cotoneaster densiflorus

Taxon classificationPlantaeRosalesRosaceae

M.W. Li, Q. Fan & W. B. Liao sp. nov.

CB30416C-B611-52C9-A3F4-DD53D957CC37

urn:lsid:ipni.org:names:77331508-1

##### Type.

**Figure 1.**
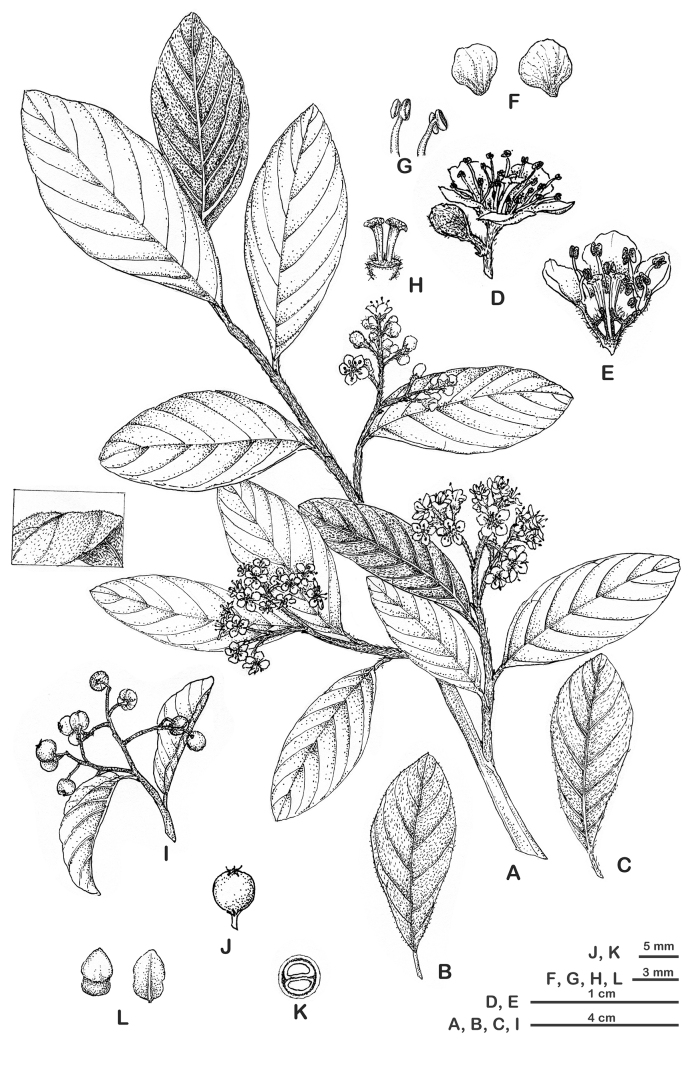
*Cotoneasterdensiflorus***A** habit **B** leaf, adaxial surface **C** leaf, abaxial surface **D** flowers **E** vertical section of flower **F** petals **G** stamens **H** styles **I** fruiting branch **J** pome **K** transverse section of pome **L** pyrenes. Illustration by Zhengmeng Yang based on living field-collected materials (*Q. Fan & M.W. Li 14925*, *M.W. Li 20230617007*).

**Figure 2.**
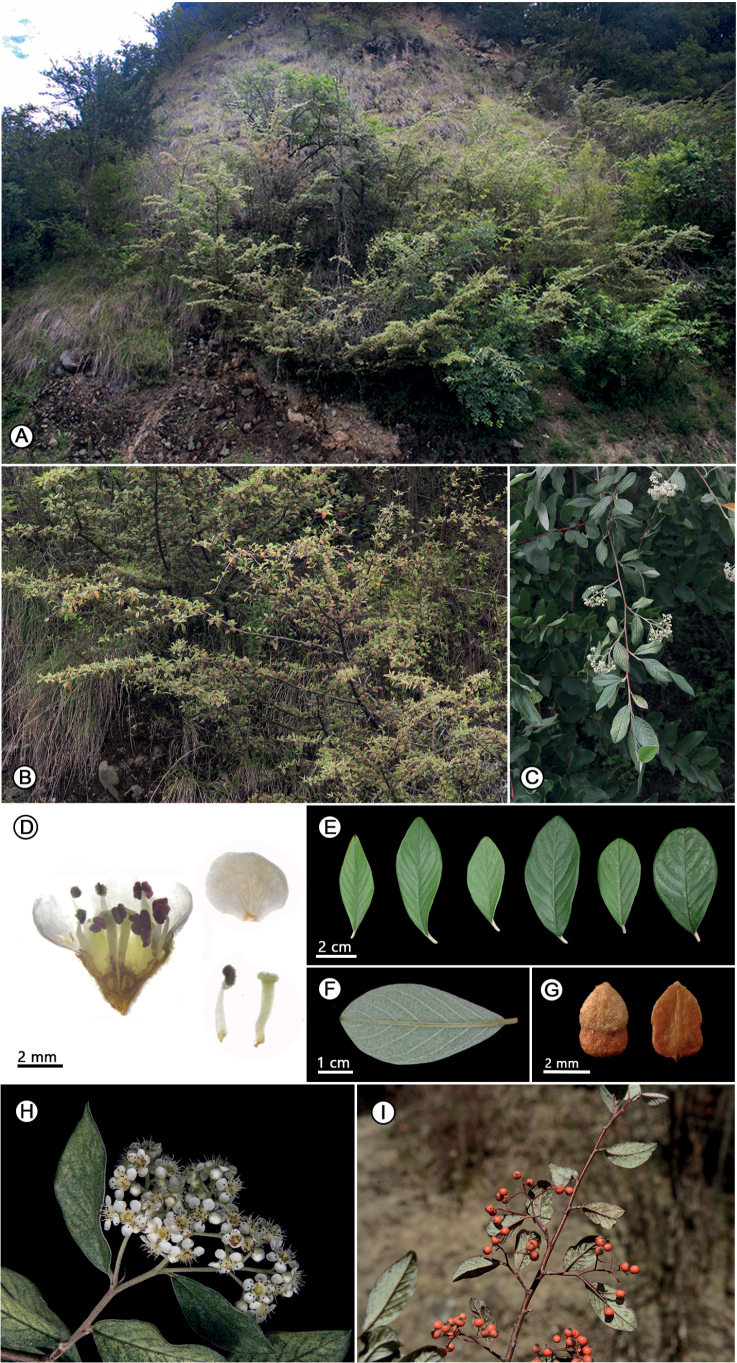
*Cotoneasterdensiflorus* sp. nov. **A** habitat **B** habit **C** branchlets **D** petal, stamen, style, and vertical section of flower **E** ovate-lanceolate to obovate shape of leaves **F** leaf, abaxial surface **G** 2 pyrenes per fruit **H** inflorescence **I** fruiting branch.

China. Sichuan Province, Baoxing County, Qiaoqi Town, Zegen Village, on the cliff of steep slopes, 30°43′N, 102°45′E, 2180 m a.s.l., 7 Dec 2016, *Q. Fan & M.W. Li 14925* (holotype: SYS; isotype: SYS) (Figs 1, 2)

##### Diagnosis.

Morphologically, *Cotoneasterdensiflorus* is similar to *C.salicifolius*, but differs in its leaf blade of ovate-lanceolate to obovate shape (vs. elliptic-oblong to ovate-lanceolate), smaller length-width ratio of 2.37 ± 0.31 (vs. 3.17 ± 0.32), slightly conduplicate (vs. not conduplicate), fewer lateral veins of 6–8 pairs (vs. 12–16 pairs), upper surface slightly rugose (vs. rugose), leaf margin plane (vs. revolute), lower surface densely grey tomentose (vs. grey tomentose, with bloom), greater corolla diameter of 7–9 mm (vs. 5–6 mm), styles 2 (vs. 2–3) and pyrenes 2 (rarely 3). Although there is a closer phylogenetic relationship between the new species and *C.rhytidophyllus*, it is easy to distinguish them by the indumentum color of branchlets, leaves and inflorescences, rugose leaf upper surface, fruit shape and pyrenes number. See Table 2, Figs 1, 2 for a detailed comparison.

**Table 2.** Diagnostic macro-morphological characteristic of *Cotoneasterdensiflorus*, *C.salicifolius* and *C.rhytidophyllus*.

	*C.densiflorus*	*C.salicifolius*	*C.rhytidophyllus*
Leaf shape	ovate-lanceolate to obovate	elliptic-oblong or ovate-lanceolate	elliptic-oblong or ovate-oblong to oblong-lanceolate
Leaf size (mm)	25–72×12–33	40–85×15–25	40–70 ×18–30
Leaf apex	acute or obtuse, rarely abruptly mucronate	acute or acuminate	acuminate, rarely acute
Leaf length-width ratio	2.37 ± 0.31	3.17 ± 0.32	3.19 ± 0.48
Leaf conduplicate state	slightly conduplicate	not conduplicate	not conduplicate
Lateral veins number (pairs)	6–8	12–16	5–8
Leaf upper surface indumentum	initially sparsely pilose	initially sparsely pilose	initially sparsely villous
Upper surface rugose state	slightly rugose	rugose	extremely rugose
Margin revolute state	plane	revolute	revolute
Leaf lower surface indumentum	densely gray tomentose	gray tomentose, with bloom	yellow tomentose-floccose
Inflorescence number of flowers	(5-)10- to 50-(61) flowers	10- to 50- flowers	10- to 40(-50) flowers
Corolla diameter (mm)	7–9	5–6	7–8
Patal indumentum	glabrous	glabrous	adaxially slightly pilose near base
Styles number	2	2–3	2–3
Fruits shape	obovoid or subglobose	subglobose	pyriform
Fruits size	5–7 mm in diam	5–7 mm in diam	4 mm in diam, 5–6 mm long
Pyrenes number	2	2–3	2–3, rarely 4

##### Description.

Evergreen shrubs, rarely semi-evergreen, up to 5 m tall, with spreading to erect branches; stems 5 cm in diameter; branchlets terete, stout, reddish-brown, initially sparsely tomentose, glabrous when old. Petiole red, robust, 4–7 mm long, tomentose; stipules linear, 4–7 mm, tomentulose, caducous; leaf blades ovate-lanceolate to obovate, 25–72 × 12–33 mm, leathery, lightly conduplicate along the mid-vein, mid-vein conspicuously raised abaxially and deeply impressed adaxially, lateral veins 6–8 pairs, rarely 5 or 9, impressed, lower surface densely grey tomentose, apex acute or obtuse, rarely abruptly mucronate, base cuneate, margin entire, plane, upper surface initially sparsely pilose, subglabrous when old, slightly rugose. Compound corymbs 25–40 mm long, 17–43 mm diam., with (5–)10– to 50–(61)-flowered per inflorescence; rachis and pedicels densely white pilose; peduncles 2–3 cm; bracts linear, tomentulose, caducous, 2–4 mm long; pedicel 2–4 mm. Flowers 7–9 mm diam.; hypanthium campanulate, abaxially densely white tomentose; sepals triangular, apex acute, pilose; petals spreading, white, glabrous, suborbicular, ca. 3–4 mm and nearly as broad, apex obtuse, base shortly clawed; stamens 20, slightly longer than or subequal to petals, anthers purple, filaments white; styles 2, free, slightly shorter than stamens; carpels 2, ovary apically pilose. The ripe pome obovoid or subglobose, 5–7 mm diam., red, sparsely pilose; 2 pyrenes per fruit.

##### Pollen morphology.

**Figure 3.**
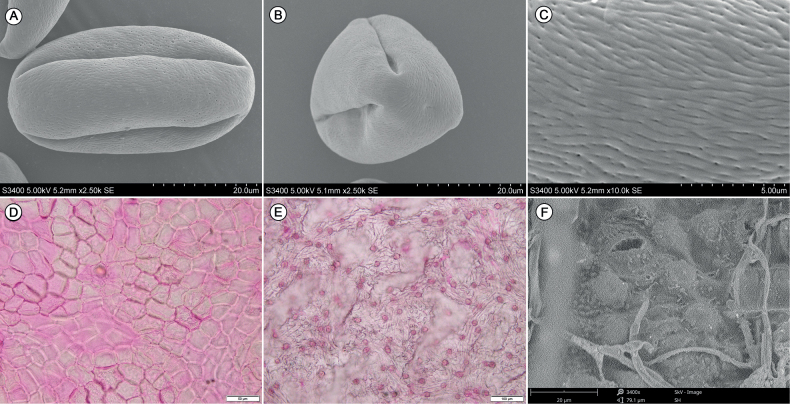
SEM micrographs of pollen grains of *Cotoneasterdensiflorus* (**A–C**), light microscope and SEM micrographs of leaf epidermis of *C.densiflorus* (**D–F**) **A** equatorial view **B** polar view **C** striate-foveolate ornamentation **D** upper epidermis **E** under epidermis **F** stomata and corneous papillae of under epidermis.

The pollen grains of *C.densiflorus* are tricolpate. Polar axis (P) = 46.15 ± 3.09 µm, equatorial axis (E) = 22.64 ± 1.28 µm, the P/E value (proportion of polar axis to equatorial axis length) = 2.05 ± 0.12. The P/E values of the new species is obviously larger than *C.salicifolius* (1.19 ± 0.05). The surface is mainly striate-foveolate ornamentation (Fig. 3, Table 3).

**Table 3.** Diagnostic micro-morphological characteristic and 2C DNA of *Cotoneasterdensiflorus*, *C.salicifolius* and *C.rhytidophyllus* (values M ± SD μm).

	*C.densiflorus*	*C.salicifolius*	*C.rhytidophyllus*
Polar axis length (µm)	46.15 ± 3.09	24.31 ± 1.07	37.39 ± 7.35
Equatorial axis length (µm)	22.64 ± 1.28	20.51 ± 0.16	18.47 ± 3.03
P/E values	2.05 ± 0.12	1.19 ± 0.05	2.02 ± 0.23
Type of leaf epidermis	type W	type I	type I
2C DNA (pg)	2.33 ± 0.19	1.57 ± 0.15	1.55 ± 0.10

##### Leaf epidermis morphology.

According to previous studies on leaf epidermis type of *Cotoneaster* species (Ding et al. 2008), the leaf epidermis of this species could be classified as type W, with irregular stomata surrounded by 4–5 corneous papillae, while *C.salicifolius* and *C.rhytidophyllus* are presented as type I, with irregular stomata surrounded by various ridges protruding from the stratum corneum (Fig. 3, Table 3).

##### Phenology.

Flowering from June to July, fruiting from November to December.

##### Etymology.

The specific epithet refers to the compact compound corymbs with (5–)10– to 50–(61)-flowered per inflorescence.

##### Distribution and habitat.

*C.densiflorus* is currently known only from the type locality, Zegen Village, Baoxing County, Sichuan Province, China. This population includes nearly 60 individuals, with about 40 densely distributed individuals and 20 scattered shrubs on a steep slope of sunny sparse forest along the National Highway at altitudes of about 2180 m a.s.l. The associated tree species include *C.dielsianus*, *Coriarianepalensis*, *Indigoferaszechuensis*, *Desmodiumelegans*, and *Elaeagnusbockii*.

##### Phylogenetic analysis.

The complete chloroplast genome of *C.densiflorus* exhibited characteristic quadripartite structure with 159,759 bp in total length, including a pair of inverted repeat (IRA and IRB) region of 26,371 bp, separated by a larger single-copy (LSC) region of 87,807 bp and a small single-copy (SSC) region of 19,210 bp with an overall GC content of 36.60%. A total of 111 unique genes were encoded, including 78 protein-coding genes (PCGs), 29 transfer RNA (tRNA) genes and four ribosomal RNA (rRNA) genes, while 17 genes duplicated in the IR regions.

**Figure 4.**
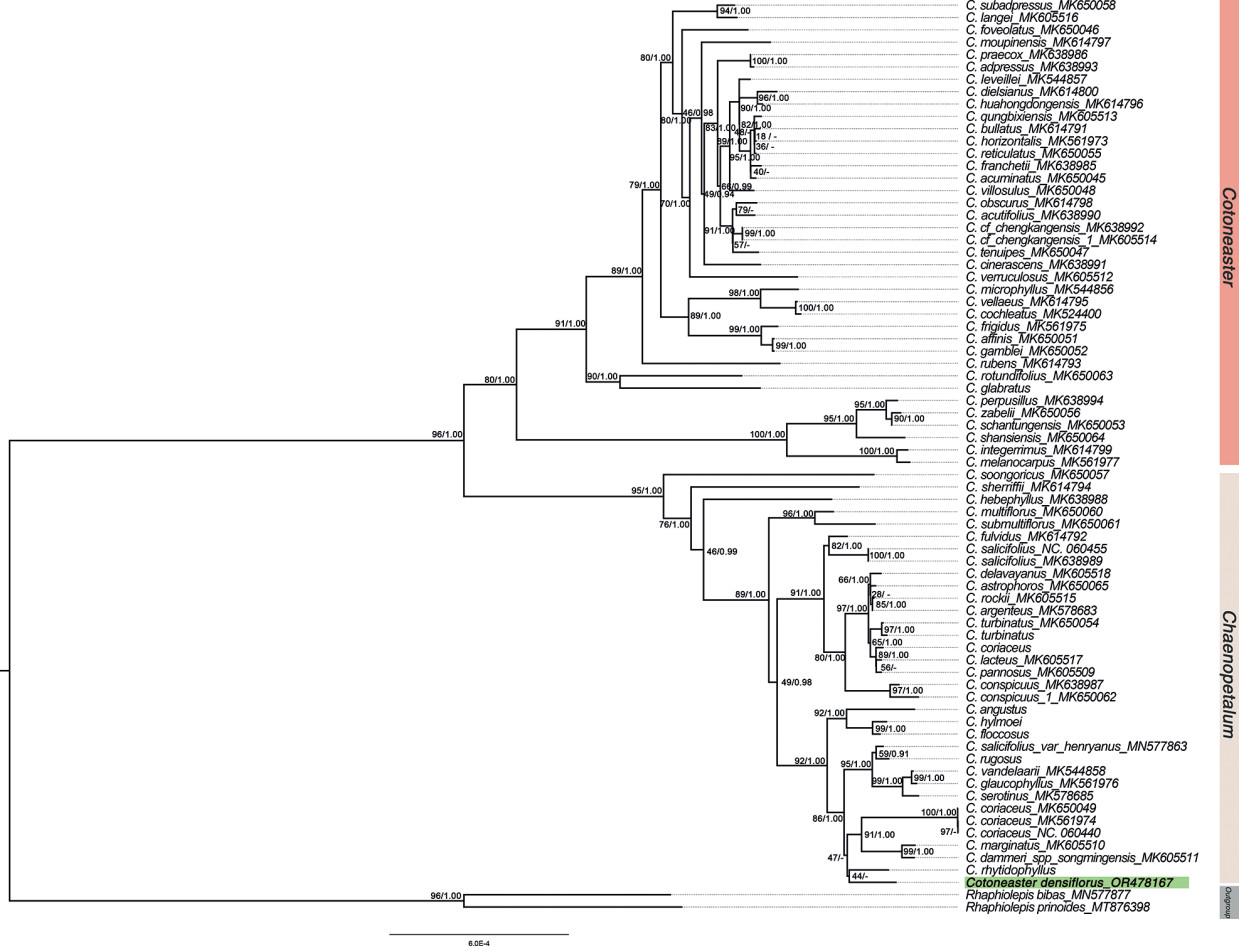
The phylogenetic tree of 65 *Cotoneaster* taxa based on 72 whole chloroplast genomes showing the position of *Cotoneasterdensiflorus* (bold and highlighted with green) in the genus. Numbers near the branches are ML and BI bootstrap values. GenBank accessions were provided after underlines.

Phylogenetic analyses constructed from 72 *Cotoneaster* chloroplast genomes resulted in the ML tree topology as shown in Table 1. Two main clades (*Cotoneaster* and *Chaenopetalum*) with well-supported values are presented in Fig. 4, which was consistent with the previous phylogenetic studies. The new species was placed in Clade *Chaenopetalum* and clustered with *C.rhytidophyllus* of Ser. Salicifolii, but with weak support values (BS = 44, BI = --).

##### Chromosome ploidy analysis.

The results of flow cytometry analysis displayed a mean genome size (2C-value) of 2.33 ± 0.19 pg for *C.densiflorus* (Table 3). Comparing with ploidy levels and genome sizes of *Cotoneaster* species reported in the previous studies (Rothleutner et al. 2016; Ksinan et al. 2021), this species was inferred as tetraploid (2n = 4x =68), while its closely-related species, *C.salicifolius* (2C-value = 1.13 ± 0.16; unpublished data) and *C.rhytidophyllus* (2C-value = 1.55 ± 0.10; unpublished data) were diploid (2n = 2x = 34) (Fryer and Hylmö 2009).

##### Conservation status.

Only one large population was found with nearly 60 mature and juvenile individuals on steep slopes about 2 km along the highway. Its habitat is affected and threatened by the violent geological, climate and artificial activities with frequent construction of highways, mud-rock flows, landslides, and even earthquakes in the last few decades. Therefore, the species could be considered as CR (Critically Endangered) status according to IUCN Red List Criteria (IUCN 2022).

##### Additional specimens examined

**(paratypes).** China. Sichuan: Baoxing County, Qiaoqi Town, Zegen Village, 30°43′N, 102°45′E, 2180 m a.s.l., 17 June 2023, *M.W. Li 20230617007* (SYS).

## ﻿Conclusions

We described and illustrated a new species of *Cotoneaster* genus (Rosaceae) in western Sichuan Province of China and provided evidence for its phylogenetic position through whole chloroplast genome data. After detailed field research, we found *Cotoneasterdensiflorus* M.W. Li, Q. Fan & W. B. Liao, sp. nov. is distributed in a narrow range of Baoxing County, which is located in the Hengduan Mountains. Only one large population of nearly 60 individuals was observed, with about 40 densely distributed individuals and 20 scattered shrubs on a steep slope of sunny sparse forest along the National Highway. Morphologically, this shrub is most similar to *C.salicifolius*, but obviously differs in leaf upper surface rugose state, margin revolute state, number of lateral veins, styles and pyrenes, pollen grains P/E values and leaf epidermis type. Our study not only enriched the diversity of *Cotoneaster* species in China, but also highlighted the importance of the basic survey of biodiversity in this area of Sichuan and the Hengduan Mountains.

## Supplementary Material

XML Treatment for Cotoneaster densiflorus

## ﻿Additional information

### Author ORCIDs

Mingwan Li https://orcid.org/0000-0002-6620-5792

Qiang Fan https://orcid.org/0000-0003-4254-6936

### Data availability

All of the data that support the findings of this study are available in the main text.
